# The Relationship Between Microbial Community and Breast Cancer

**DOI:** 10.3389/fcimb.2022.849022

**Published:** 2022-06-16

**Authors:** Xuelian Song, Changran Wei, Xiangqi Li

**Affiliations:** ^1^ Department of The Graduate Student, Shandong First Medical University, Tai’an, China; ^2^ Department of The First Clinical Medical School, Shandong University of Traditional Chinese Medicine, Jinan, China; ^3^ Department of Breast Surgery, The Second Affiliated Hospital of Shandong First Medical University, Tai’an, China

**Keywords:** breast cancer, microbial community, local microenvironment, immunity, relationship

## Abstract

Breast cancer (BC) is the most common cancer in women and the leading cause of cancer-related deaths in women worldwide. Recent research studies have shown that the intestinal flora is related to the occurrence and progression of BC. Notably, some evidence identifies a unique microbial community in breast tissue, a site previously thought to be sterile. In addition, breast tumors have their own specific microbial community, distinct from normal mammary gland tissue, and all of them may result from intestinal flora. Some microbial community in breast tissue may lead to the occurrence and development of BC. This review focuses on the relationship between the microbial community and breast cancer, which will lay a solid theoretical foundation for further understanding the local microenvironment of BC and developing effective targeted therapeutic drugs.

## Introduction

Though accounting only for 2-7% of biomass owing to the miniscule size of microbes, the human microbiome encodes for 100-fold more genes than the human genome indicating an important role in human health (Bhatt et al., 2017). Microbiota and host maintain a dynamic equilibrium referred to as eubiosis that actively influences many physiological processes and is generally beneficial to the host. However, a state of disequilibrium or dysbiosis may evolve contributing to various disease states (Parida et al., 2021). One of the most major achievements in the microbiome field was identified that the role of microbes in the gastrointestinal tract (Turnbaugh et al., 2007; Peterson et al., 2009; Proctor et al., 2019). More recent developments show the existence of microbiota in other body sites, initially considered ‘sterile’, such as breast (Urbaniak et al., 2014a; O'Connor et al., 2018).

Presently, breast cancer (BC) is the most common malignant tumour and the most important health burden among women worldwide. Recently, statistics have shown that the incidence of BC in various countries around the world is increasing at an accelerated rate, and the affected population is becoming younger (Siegel et al., 2020). With the development of imaging technology, surgery and medical treatments, the diagnosis and treatment of BC have improved. The survival rate of BC patients is increased, and the recurrence rate and mortality rate have decreased correspondingly but remain high (Desantis et al., 2019). Therefore, exploring the aetiology and pathogenesis of BC is still a top priority. BC is a complex disease that is influenced by many factors, including genetic factors, diet, obesity, endocrine hormone levels and others. Recent research studies have shown that the microbial community is related to the occurrence and progression of BC (Xue et al., 2018).

Some evidence identifies a unique microbial community in breast tissue, a site previously thought to be sterile (O'Connor et al., 2018). In addition, breast tumours have their own specific microbial community, distinct from normal mammary gland tissue (Donnet-Hughes et al., 2010; Chiba et al., 2020). Some microbial community in breast tissue may lead to the occurrence and development of BC. In addition to breast microbiota, some studies have shown that gut microbiota may also influence breast cancer. Furthermore, microbial signatures may differ between different breast cancer patients. For example, an analysis of fecal microbiota shows that postmenopausal women with breast cancer harbor compositionally different gut microbiota than healthy volunteers and exhibit enrichment of several bacterial species (Goedert et al., 2015a; Zhu et al., 2018). In addition, breast cancer is a heterogeneous disease with multiple subtypes and interestingly, microbial signatures may differ between the subtypes (Banerjee et al., 2018).

Furthermore, breast cancer cells are able to repurpose pre-existing metabolic symbiosis, leading to profound alterations in the local microenvironment (Nunes and Serpa, 2020), thus further promoting the development of BC. There are encouraging signs that the breast tumour microbial community is modified by therapy and affects the molecular signaling pathway and the internal environment (Chiba et al., 2020), thus achieving a therapeutic effect. In summary, this review focuses on the relationship between the microbial community and tissue and points out the carcinogenic mechanism of the microbial community in the occurrence and development of BC, as well as the treatment methods, which will lay a solid theoretical foundation for further understanding the carcinogenic mechanism of BC and developing effective targeted therapeutic drugs.

## Microbial Community in Breast Tissue

Most studies have focused on the intestinal microflora by exploring the relationship between microorganisms and cancer (Tsilimigras et al., 2017), but the increasing understanding of the existence of microorganisms in and adjacent to tumour sites has also brought some new discoveries, which are useful for revealing the carcinogenic mechanism of microflora and the related microenvironment (Chen et al., 2017). Considering the different effects that the microbial community has in distinct organs, recent studies have focused on examining colonizing bacteria in breast tissue. Breast cancer is one of the earliest and most intensively studied diseases using genomic technology (Xuan et al., 2014), but it is only recently that the existence of microorganisms in breast tissue and the potential role of mammary duct microbial community have been explored (Urbaniak et al., 2014b; Chan et al., 2016). In this regard, specific microbial community have been identified in breast milk (Arthur and Jobin, 2013), and several authors postulated that bacteria are capable of using the nipple to gain access to the breast ducts and create a specific microbial community in the breast. This is not surprising considering that skin and oral bacteria have access to the breast ducts through the nipple (Ramsay et al., 2004), we guess that means that breastfeeding could play an important role. But interestingly, recent studies have suggested that their origin is the mother’s gastrointestinal tract (Donnet-Hughes et al., 2010). Now, let us look closer at what microbial community are in the breast tissue (**Table 1**).

**Table 1:** Summary of studies about the microbial community in breast tissue.

Tissue Samples	Test Group	Main Methodology	Microbial Expression	Reference
Breast tissue	43 Canadian women (11 with benign tumors, 27 cancerous tumors and 5 healthy individuals) and 38 Irish women (33 women with BC and 5 healthy individuals)	V6 16S rRNA sequencing (Ion Torrent) Pipeline: UCLUST	↑*Proteobacteria* and *Firmicutes* in breast tissue. ↑*Bacillus* (11.4%) and *Acinetobacter* (10%) in Canadian women. ↑*Enterobacteriaceae* (30.8%) and Staphylococcus (12.7%) in Irish women. ↑*Escherichia coli* in BC tissue.	(Urbaniak et al., 2014b)
Breast tumor tissue and its paired normal adjacent tissue	20 patients ER+ BC	Pyrosequencing V4 16S rDNA Pipeline: QIIME	↑*Proteobacteria, Firmicutes, Actinobacteria, Bacteroidetes* and *Verrucomicrobia* (96.6%) in breast tissue. ↑*Methylobacterium radiotolerans* in BC tissue. ↑*Sphingomonas yanoikuyae* in paired normal tissue.	(Xuan et al., 2014)
Breast tissue	668 tumor tissues (HER2+, ER+, TNC) and 72 normal adjacent tissues from The Cancer Genome Atlas (TCGA)	V3-V5 16S rRNA amplified sequencing data	↑*Proteobacteria, Actinobacteria* and *Firmicutes* in breast tissues. ↑*Proteobacteria, Mycobacterium fortuitum* and *Mycobacterium phlei* in BC samples. ↑*Actinobacteria* in normal adjacent tissue.	(Thompson et al., 2017)
Breast tissue	57 women with invasive breast carcinoma and 21 healthy women	V3-V4 16S rRNA sequencing (Illumina) Pipeline: UCLUST	↓*Methylobacterium* and ↑*Corynebacterium*, *Staphylococcus*, *Actinomyces* and *Propionibacteriaceae* in patients with invasive breast carcinoma compared to healthy individuals.	(Wang et al., 2017)
Breast tissue	16 Mediterranean patients with BC (12 samples were collected from core needle biopsies (CNB) and 7 from surgical excision biopsies (SEB); 3 patients were processed with both procedure)	V3 16S-rRNA gene amplicons sequencing (Ion Torrent)	↑*Ralstonia* in breast tissue. No significant differences between healthy adjacent breast tissues and BC tissues.	(Constantini et al., 2018)
Breast tissue	22 Chinese patients with benign tumor and 72 malignant BC patients	V1-V2 16S rRNA sequencing (Illumina HiSeq)	↑*Propionicimonas, Micrococcaceae, Caulobacteraceae, Rhodobacteraceae, Nocardioidaceae and Methylobacteriaceae*, in BC tissues (ethno-specific). ↓*Bacteroidaceae* and ↑ *Agrococcus* are related with malignancy	(Meng et al., 2018)
Breast tissue	58 women: 13 benign, 45 cancerous tumors and 23 healthy women	V6 16S rRNA gene sequencing (Illumina MiSeq) Pipeline: QIIME	↑*Bacillus, Enterobacteriaceae, Staphylococcus, Comamondaceae* and *Bacteroidetes* and ↓ *Prevotella*, *Lactococcus*, *Streptococcus*, *Corynebacterium* and *Staphylococcus* in BC patients compared to healthy controls.	(Urbaniak et al., 2016)
Nipple aspirate fluid (NAF) and aerolar breast skin	25 women with breast ductal cancer and 23 healthy women	V4 16S rRNA gene sequencing (Illumina MiSeq) Pipeline: Mothur	↑*Alistipes* and ↓ unclassified genus of the *Sphingomonadaceae* family in NAF from women with BC compared to healthy controls.	(Chan et al., 2016)
Breast tissue	100 women with triple negative BC (TNBC), 17 matched controls and 20 non-matched controls	PathoChip array	↑*Brevundimonas diminuta, Arcanobacterium haemolyticum*, *Peptoniphilus indolicus, Prevotella nigrescens, Propiniobacterium jensenii* and *Capnocytophaga canimorsus* in TNBC. Among virus, ↑ *Herpesviridae*, *Retroviridae, Parapoxviridae, Polyomaviridae*, *Papillomaviridae* in TNBC.	(Banerjee et al., 2015)
Breast tissue and breast skin	28 women undergoing non-mastectomy breast surgery: 13 benign breast disease and 15 invasive BC (100% ER/PR+ and 29% HER2+)	V3-V5 16S rDNA hypervariable taq sequencing (Illumina MiSeq) Pipeline: IM-TORNADO	↑*Fusobacterium*, *Atopobium*, *Gluconacetobacter*, *Hydrogenophaga* and *Lactobacillus* in BC tissue compared to healthy breast tissue.	(Hieken et al., 2016)
Breast tissue	20 normal breast tissue and 148 BC tissue (50 ER or PR+, 34 HER2+, 24 TP and 40 TN)	Pathochips array	↑*Proteobacteria* and ↑*Actinomyces* in the four BC subtypes studied.	(Banerjee et al., 2018)
Snap-frozen breast tumor tissue	15 women with BC who were treated with neoadjuvant chemotherapy, 18 women with no prior therapy at time of surgery and 9 women who had tumor recurrence	V4 16S rRNA amplicon sequencing (Illumina Miseq) Pipelinee: Mothur (v.1.39.5) Microarray for confirmation	↑*Pseudomonas* spp. in BC tissue after neoadjuvant chemotherapy. ↓*Prevotella* in the tumor tissue from non-treated patients. ↑*Brevundimonas* and *Staphylococc*us in the primary breast tumors in patients developing distant metastases.	(Chiba et al., 2020)

### In Normal Breast Tissue

The breast is composed of epithelial, interstitial and mucosal immune systems, which constitute a complex microenvironment (Going and Moffat, 2004). Since the development of the mucosal immune system is the direct result of microbial exposure, inflammation is partly related to the changes in the microenvironment induced by bacterial infections (Schwabe and Jobin, 2013; Garrett, 2015), so the presence of immune effects in the complex microenvironment of the breast indicates the breast microbial community. At present, there are some predominant microbial community in the normal breast tissue, such as *Proteobacteria* and *Firmicutes* (Urbaniak et al., 2014b), *Sphingomonas yanoikuyae* (Xuan et al., 2014), *Actinobacteria* (Thompson et al., 2017), *Methylobacterium* (Wang et al., 2017), *Ralstonia* (Constantini et al., 2018), *Bacteroidaceae* (Meng et al., 2018), *Prevotella*, *Lactococcus*, *Streptococcus*, *Corynebacterium*, *Staphylococcus* (Urbaniak et al., 2016), unclassified genus of the *Sphingomonadaceae* family in NAF (Chan et al., 2016), and others, which can be seen in the **Table 1**.

### In Breast Tumour Tissue

Compared with the normal breast tissue, the microbial spectrum in breast tissues of breast cancer patients is significantly different. Among them, proteobacteria are the most abundant species in normal breast tissue (Mani, 2017). Generally, microbial community enriched in malignant tumour tissues include *Proteobacteria, Firmicutes, Escherichia coli, Methylobacterium radiotolerans, Mycobacterium fortuitum, Mycobacterium phlei, Corynebacterium, Staphylococcus, Actinomyces, Propionibacteriaceae, Propionicimonas, Micrococcaceae, Caulobacteraceae, Rhodobacteraceae, Nocardioidaceae, Methylobacteriaceae, Bacillus, Enterobacteriaceae, Comamondaceae, Bacteroidetes, Alistipes, Brevundimonas diminuta, Arcanobacterium haemolyticum, Peptoniphilus indolicus, Prevotella nigrescens, Propiniobacterium jensenii, Capnocytophaga canimorsus, Fusobacterium, Atopobium, Gluconacetobacter, Hydrogenophaga, Lactobacillus*, and some others (Xuan et al., 2014; Urbaniak et al., 2014b; Banerjee et al., 2015; Chan et al., 2016; Urbaniak et al., 2016; Hieken et al., 2016; Thompson et al., 2017; Wang et al., 2017; Constantini et al., 2018; Meng et al., 2018), which are different from the normal tissue. Notably, *Pseudomonas* spp. in BC tissue increase after neoadjuvant chemotherapy. Compared with tumour tissue from treated patients, Prevotella are decreased in non-treated patients with BC.

Furthermore, focusing on the shift in microbial community composition in breast tissue from patients with disease compared to normal breast tissue, researchers have identified the presence of Bacteroides fragilis in cancerous breasts. Mammary gland and gut colonization with enterotoxigenic Bacteroides fragilis (ETBF), which secretes B. fragilis toxin (BFT), rapidly induces epithelial hyperplasia in the mammary gland. Breast cancer cells exposed to BFT exhibit ‘BFT-memory’ from the initial exposure. Intriguingly, gut or breast duct colonization with ETBF strongly induces the growth and metastatic progression of tumour cells implanted in mammary ducts in contrast to non-toxigenic Bacteroides fragilis. This work sheds light on the oncogenic impact of the pro-carcinogenic colon bacterium ETBF on breast cancer progression (Parida et al., 2021).

In fact, breast cancer is a heterogeneous disease. Using a whole genome and transcriptome amplification and a pan-pathogen microarray (PathoChip) strategy, Banerjee’s research group investigated the diversity of the microbiome in the four major types of breast cancer: endocrine receptor (ER) positive, triple positive, Her2 positive and triple negative breast cancers (Banerjee et al., 2018). The microbial communities for each breast cancer molecular subtype shown in **Table 2**.

**Table 2:** Microbial communities in different molecular subtypes of breast cancer.

Molecular Subtypes of BC	Microbial Community			
	Bacteria	Viruses	Parasites	Fungus
ER+	*Arcanobacterium, Bifidobacterium, Cardiobacterium, Citrobacter, Escherichia*		*Brugia, Paragonimus*	*Filobasidilla, Mucor, Trichophyton*
triple positive	*Bordetella, Campylobacter, Chlamydia, Chlamydophila, Legionella, Pasteurella*	*Birnaviridae, Hepeviridae*	*Ancylostoma, Angiostrongylus, Echinococcus, Sarcocystis, Trichomonas, Trichostrongylus*	*Penicillium*
Her2+	*Streptococcus*	*Nodaviridae*	*Balamuthia*	*Epidermophyton, Fonsecaea, Pseudallescheria*
TNBC	*Aerococcus, Arcobacter, Geobacillus, Orientia, Rothia*		*Centrocestus, Contracaecum, Leishmania, Necator, Onchocerca, Toxocara, Trichinella, Trichuris*	*Alternaria, Malassezia, Piedraia, Rhizomucor*

In addition, some researchers have compared the microbial community profiles in different histological grades of malignant tumour tissues and found that with the development of tumours, the relative abundance of the Bacteroides family decreases, and the relative abundance of Agrococcus increases (Mani, 2017). The specific correlation between these potential microbial markers and advanced disease may have broad significance in the diagnosis and staging of breast cancer.

## Influence of Gut Microbial Community on Breast Cancer

Until now, in addition to the microbial community in breast tissue, evidence from animal experiments also confirmed the relationship between the gut microbial community and breast tissue. The gut microbial community may have an effect on the occurrence and development of BC, and possible mechanisms include estrogen metabolism, diet and obesity, inflammation, immune regulation and bacterial toxin production (Yang JQ et al., 2017).

### Estrogen Metabolism

In addition to traditional risk factors such as family history, age, and atypical proliferative breast disease, elevated levels of endogenous or circulating estrogen are directly associated with an increased risk of breast cancer in postmenopausal women (Dallal et al., 2014). Studies have suggested that the gut microbial community may be associated with BC through a response to estrogen metabolism (Goedert et al., 2015b).

**Figure 1:**
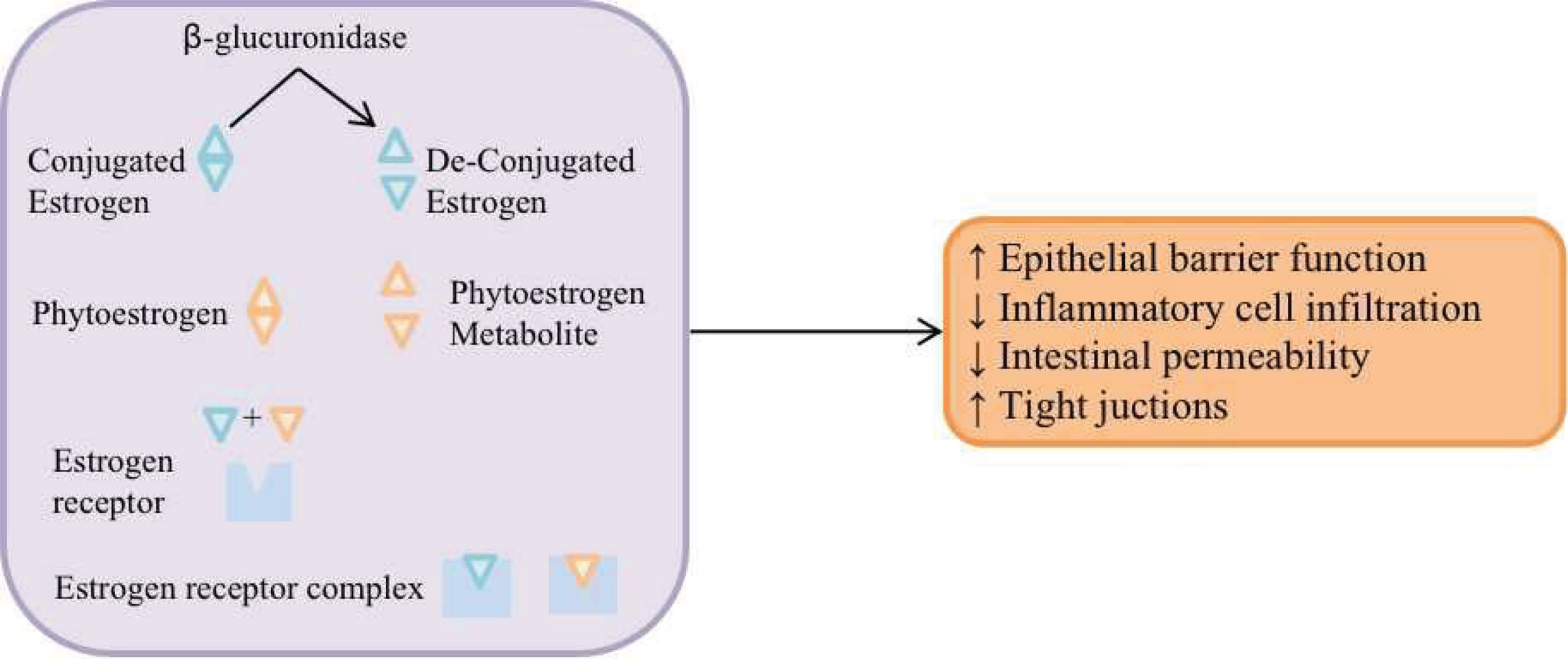
The gut microbial community regulates estrogens through secretion of β-glucuronidase. β-glucuronidase de-conjugates estrogen to enable the binding to estrogen receptors, and then, regulates the intestinal function and micro-environment and increase the breast cancer risk.

The gut microbial community regulates estrogens through secretion of β-glucuronidase. β-glucuronidase de-conjugates estrogen to enable the binding to estrogen receptors (Alizadehmohajer et al., 2020). And then, the estrogen receptor complex could regulate the intestinal function and micro-environment and increase the breast cancer risk (**Figure 1**). In addition, in postmenopausal women, some circulating estrogens in the body are determined by the estrogens involved in the liver-gut circulation, and some gut bacteria are more likely to enter the liver-gut circulation by binding to the estrogens that are excreted in the gut bile; therefore, estrogen and estrogen-like substance concentrations in the body may increase the incidence of BC (Yang JQ et al., 2017).

Notably, dietary estrogens, or phytestrogens, are exogenous estrogens that compete with endogenous estrogen receptor 1 (ESR1). However, the difference is that phytestrogens can reduce the incidence of breast cancer. Enterolactone is a phytestrogen that is the result of the fermentation of lignans by intestinal bacteria. Some experts believe that enterolactone may be used as a drug to inhibit the proliferation of BC cells (Shapira et al., 2013).

### Diet and Obesity

Some potential risk factors for BC, such as endogenous and exogenous substance metabolism and obesity status, are related to gut microbial community (Attraplsi et al., 2013). Diet is an important external factor that affects the gut microbial community (Yang JQ et al., 2017), and people who eat different diets over a long period of time have very different microbiomes (Ou et al., 2013). Early great milestone-style work by Doll and Peto suggested that diet is responsible for approximately 35% of cancers (Doll and Peto, 1981). It has been demonstrated that impaired absorption of nutrients can alter the morphology of the gut in non-reproductive mice, leading to a reduction in the number and function of immune cells and a reduction in the production of antimicrobial peptides and immunoglobulins (Round and Mazmanian, 2009), thus indirectly promoting the occurrence of BC. Under a normal diet, intestinal flora can regulate the content of lipopolysaccharide and the production of short-chain fatty acids, directly or indirectly affecting the process of lipid metabolism and affecting the energy balance and body weight of individuals (Velagapudi et al., 2010). The gut microbial community is involved in the occurrence and development of obesity mainly by promoting the production of short-chain fatty acids, inhibiting fasting-induced adipose factor (FIAF), mediating chronic mild inflammatory reactions and inhibiting fatty acid oxidation (Fandriks, 2017). A long-term high-sugar and high-fat diet will change the distribution of gut microbial community, thus leading to the occurrence and development of BC.

### Inflammation and the Immune Response

**Figure 2:**
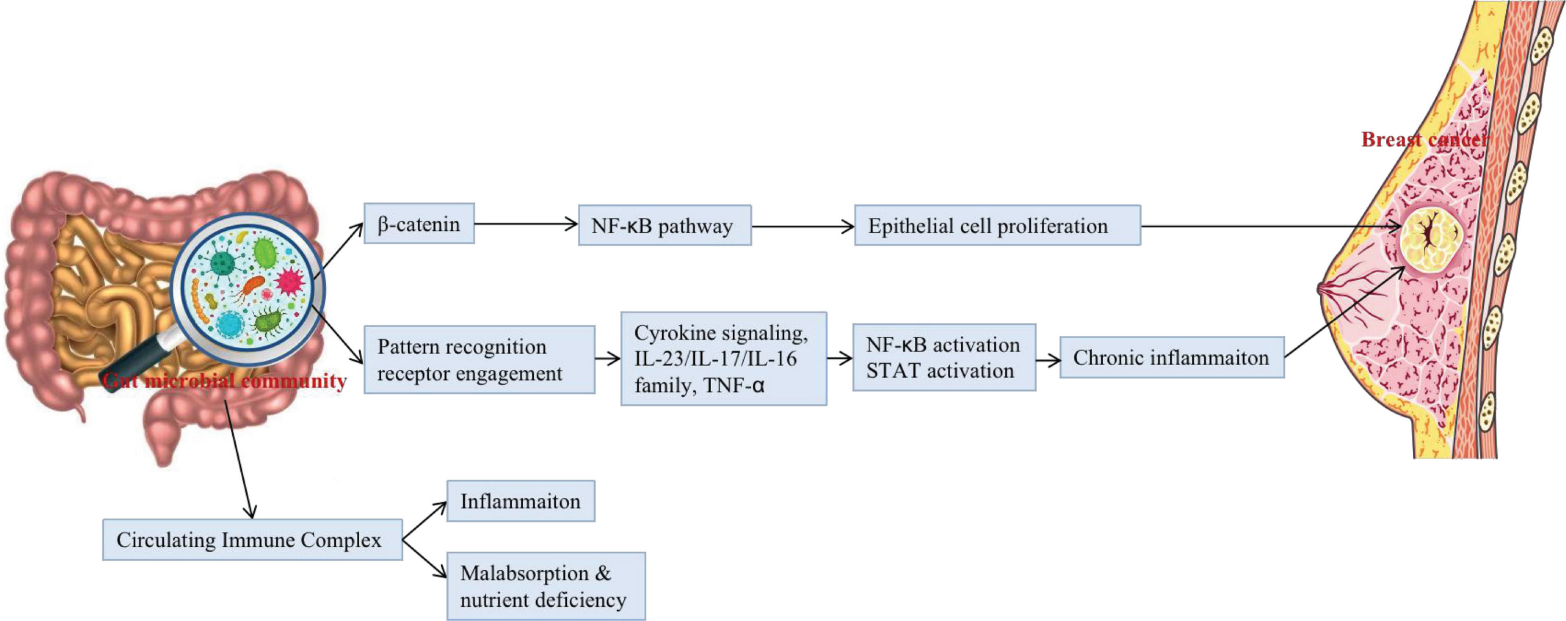
The potential mechanisms that gut microbial community may be associated with the occurrence and development of BC.

It is known that the expansion of dysbacteriosis in the gut and the extravasation of microbial products can lead to a chronic pro-inflammatory state, which negatively affects the immune system and is unconducive to the elimination of mutant and senescent cells, thus promoting the growth of tumours (Biragyn and Ferrucci, 2018). Changes in intestinal microflora are associated with the development of both intra- and extra-intestinal cancers through the initiation of chronic inflammation and changes in the microenvironment and metabolism (Dapito et al., 2012). Notably, this state may be irreversible (**Figure 2**).

The gut microbial community may be associated with the occurrence and development of BC by influencing T cells, neutrophils and some related inflammatory factors (Yang JQ et al., 2017). Rutkowski et al. confirmed that in breast cancer patients, there is an interaction between symbiotic bacteria, IL-6, and neutrophils (Rutkowski et al., 2015). Such findings have led researchers to wonder whether downregulation of inflammatory factors and neutrophils could reduce the risk of BC. Studies by Poutahidis have shown that oral administration of Lactobacillus reuteri isolated from human milk to mice can reduce the expression of inflammatory factors (Poutahidis et al., 2013; Poutahidis et al., 2013). Subsequent experiments have shown that the risk of BC could be reduced by downregulating the expression of inflammatory factors in mice.

### Bacterial Toxin Production

Studies have shown that some bacterial in humans can release some bacterial toxins, such as the enterotoxin released by *Proteobacteria*, inducing inflammatory bowel disease such as colitis, and damage the intestinal barrier leading to translocation of non-pathogenic bacteria, thus affecting the stability of the immune system and inducing oncogenic-related immune responses that lead to the development of intestinal or extra-intestinal cancers (Rubin et al., 2012), such as BC. Bacterial toxins bind specifically to pattern recognition receptor receptors, such as Toll-like receptors and Nod-like receptors; activate corresponding signalling pathways that cause the expression of chemokines, inflammatory factors, and antimicrobial peptides; promote the proliferation of tumour cells; inhibit the apoptosis of tumour cells; and inhibit the anti-tumour immune response, thus promoting the invasion and metastasis of tumour cells, tumour angiogenesis and other malignant biological behaviours (Kinnebrew and Pamer, 2012). Moreover, some bacterial species may contribute to chronic inflammatory disease by increasing reactive oxygen species production that may eventually mediate genotoxicity. Carcinogenesis can also be modulated by releasing different bacterial toxins that cause DNA damage. As bacteria cross the epithelial barrier, they can directly insert the toxins into the cell of the host. Various bacterial toxins such as Bacillus fragilis, colibactin, and cytolethal cause a carcinogenic cell responses; specifically against DNA damage (Raza et al., 2019).

## Clinical Significance

A new role for microflora is as biomarkers. Biomarkers are indicators of the presence or severity of disease. Several studies have reported associations between bacterial markers and clinical or treatment outcomes. Because the intestinal microflora is a rich source of potential biomarkers (Wong and Yu, 2019), the gut microbial community make it possible to predict the responses to disease levels and to treatment. Currently, there is growing evidence that microbial-host interactions may influence or serve as biomarkers for the pathogenesis of BC.

### Preventive Effect

The composition of the gut microbiome is not set in stone and instead depends on internal and external factors, such as diet, lifestyle, infection, ageing, antibiotics, activation of the immune response, and IGA produced by B cells. Therefore, the gut microbial community can be regulated to play a preventive role in BC. We can do some beneficial things in our daily life, such as consuming a healthy diet, balancing regular work and rest, and performing the necessary amount of exercise.

Furthermore, several *in vitro* and *in vivo* studies investigated the effect of probiotics on BC; for instance, significant inhibition of cell proliferation, induction of apoptosis, and cell cycle arrest of Enterococcus faecalis and Staphylococcus hominis are proved (Hassan et al., 2016). Lakritz et al. studied two groups of mice: a group manipulated to develop human breast tumors and the other group fed by a Western-style diet (high fat and sugar, low vitamin D3, vitamin C, and fiber) to develop mammary tumors. The two groups were treated with oral intake of probiotic lactic acid microbes. The results showed that the probiotic Lactobacillus reuteri inhibited early-stage carcinogenesis and raised breast cell sensitivity to apoptosis (Lakritz et al., 2014).

Additionally, it was confirmed that oral administration of L. acidophilus represents anticancer activity in mice bearing breast tumors (Yazdi et al., 2010). Another *in vivo* study showed that drinking milk fermented with Lactobacillus helveticus R389 elevated IL- 10 and decreased IL-6 levels both in serum and mammary cells of mice, which lead to breast tumor cell inhibition (Alejandra et al., 2005). Moreover, anticancer effects of probiotics on cancer cell lines are well gathered in the review by Mendoza et al. They showed anti-proliferative activity, apoptosis, cytotoxicity, and cell cycle arrest of probiotics (Mendoza, 2019). Long-term exposure to probiotics such as L. casei Shirota and soy isoflavones in Japanese females demonstrated their chemopreventive effect on cancer development (Toi et al., 2013). Many experts also believe that human symbiotic microbes are more flexible and manoeuvrable than genomes, so we can try to regulate the gut microbial community to prevent tumours. In addition, it may be possible to prevent cancer by using drugs that target bacterial inflammation or genetic toxins (Garrett, 2015).

### Therapeutic Effect

Altering the microbial community can affect the growth of cancer and prevent its recurrence. Previous studies have found that injecting Lactobacillus acidophilus into mice with breast tumours alters the production of cytokines and the growth of tumours, possibly altering microbes in the gut, tumour or elsewhere (Maroof et al., 2012). Recent studies in mouse models of colon cancer suggest that oral probiotic supplements containing Lactobacillus helveticus may reduce the production of IL-17-producing T cells by altering the gut microbiome, thus reducing the proliferation of tumour cells (Rong et al., 2019). Taking into account the epidemiological similarities between colon cancer and BC and referring to the hypothesis of gut microbial community and the aetiology of BC, it is important to think deeply whether interfering with the gut microbial community has an effect on BC treatment.

In addition, microbiome regulation may be used as an adjunct to standard cancer therapy (Mani, 2017). Studies have shown that the regulation of microbial community during treatment may help to mitigate the adverse effects of cancer treatment (Montassier et al., 2015). For example, cisplatin, a platinum-based chemotherapy drug, can lead to destruction of the intestinal epithelial barrier and translocation of intestinal bacteria. Cisplatin was found to destroy the intestinal epithelium and change the gut microbial community in a mouse tumour model, thus leading to a series of adverse reactions. However, interestingly, these reactions could be eliminated by administering medicine with Micrococcus or faecal granules (Perales-Puchalt et al., 2018). In addition, the selective use of specific subgroups of the gut microbial community in chemotherapy can promote anti-tumour immunity. For example, fragile bacteriocin is a key factor in targeting the anti-tumour effect of antibodies against cytotoxic T lymphocyte-associated protein 4 (CTLA-4) (Vetizou et al., 2015). Similarly, the relationship between Bifidobacterium and anti-programmed death ligand receptor (PD-L1)/anti-programmed death receptor (PD-1) therapy has been demonstrated (Sivan et al., 2015; Fessler et al., 2019; Strouse et al., 2019). Dysbiosis, prevalent in non-responders to anti-PD-1 therapy, may cause inflammation and the arrest of T cell differentiation into CD8+ effector cells, and has been associated with a significant reduction in the proportion of Sphingomonas. Oral Bifidobacterium can increase tumor cell control and contributes to interferon (IFN)-γ production by CD8+ tumor-specific T cells, and further increases the activation of intratumoral dendritic cells to improve anti-PD-L1 efficacy. What’s more, it is worth mentioning that the intestinal injury and alteration of microflora caused by cisplatin may be a part of its anti-tumour effect (Gui et al., 2015).

Furthermore, microbiome engineering may open new horizons in prevention, diagnosis, and treatment of cancer. As mentioned above, alterations in gut bacterial community may increase the risk of cancer. Therefore, designing antibiotics that target a particular spectrum of the microbiome might help regulate the gastrointestinal microbiome as a possible way to reduce the BC risk (Yang J et al., 2017). It may occur through changes in the activation of signaling pathways as well as the innate and acquired immune responses (Zahra et al., 2020). Engineered probiotics might be useful in targeting these signaling pathways. But, the diversity of bacterial community may make it challenging to developing the antibiotics and identify the cancer.

## Discussion and Conclusion

With the discovery and exploration of microbial community, people not only understand the existence of specific microbial community in the breast tissue but also realize the correlation between BC and microbial community in the breast tissue or in the gut. However, notably, regarding the local microflora of breast tissue, not all of them play a certain role in the occurrence and development of BC, and some may have no significance to the development of diseases, such as Ralstonia.

In addition, it is not clear from where the local microflora of the breast actually originates. It is also not clear what the relationship between the microbial community of the breast tissue and the microbial community of the gut is. Currently, there may be three hypotheses as follows: 1) the bacteria enter the ducts of the breast by the nipple and produce a specific microbial community in the breast tissue; 2) bacterial translocation from the gut microbial community; and 3) bacterial invasion originates from the mother’s intestinal mucosa. The first assumption is the one that most people accept. For the second hypothesis, the related reason is that the bacteria can spread through the blood and migrate to breast tissue. Indeed, studies have shown that, especially during lactation, cells from gut-associated lymphoid tissue travel to the breast *via* the lymphatics and peripheral blood (Donnet-Hughes et al., 2010). Furthermore, in a mouse model, increased bacterial translocation from the gut during pregnancy and lactation and the presence of bacterially loaded dendritic cells in lactating breast tissue have been shown (Donnet-Hughes et al., 2010). Therefore, some researchers proposed the third hypothesis:bacterial invasion originates from the mother’s intestinal mucosa.

New findings are always worth exploring, so when a hypothesis is raised, it also raises some questions that need to be considered: 1) Is BC caused by bacterial translocation or invasion? If so, is it possible to intervene in infants during the breastfeeding phase? 2) Is it possible to change the local microbial community of the breast tissue by altering the gut microbial community? 3) In addition to the carcinogenicity of gut microbial community, can microbial community play a role in inhibiting BC? Which microbial community are involved, and how can we increase their number?

In addition, chemotherapy drugs for BC, such as paclitaxel, have previously been thought to work by acting directly on tumour cells. However, recently, paclitaxel was found to inhibit tumour metabolism by modifying the gut microbial community (Su et al., 2019). Therefore, can paclitaxel also change the local microenvironment of breast tumour tissue? In other words, is its therapeutic effect on tumours due to changes in local microorganisms, which thus allows the dominant bacteria to play a role in killing tumours?

At present, research on the microbial community related to breast tissue is still at the initial stage. The relatively definite finding is that there are some specific microbial community in the breast tissue. However, whether these bacteria are related to the development of BC is not clear. In addition, whether the microbial community in the breast tissue is the cause or a result of BC needs to be considered. Interestingly, some studies by Urbaniak et al. have shown that bacterial communities do not differ between tumour tissue and normal adjacent tissue at either the population or individual level (Fernández et al., 2018). So what is the point of microbial community in breast tissue?

In conclusion, the relationship between microbial community and breast tissue is a new field of mammary gland research. Notably, the microbial community is a double-edged sword that regulates the local and systemic immune responses. On the one hand, it can lead to the occurrence and development of cancer. On the other hand, microbial community is of great significance in preventing the occurrence and development of cancer. Therefore, in-depth study of the microbial community is necessary. Perhaps in the future, microbial community will provide an unprecedented treatment for BC, especially TNBC, which currently lacks an efficient method to cure.

## Author Contributions

XS wrote this manuscript. CW and XL revised the manuscript. All authors contributed to the review and approved the submitted version.

## Funding

The present study was supported by the National Natural Science Foundation of China (grant no. 81473687), the Academic Promotion Program of Shandong First Medical University (grant no. 2019QL017), the Natural Science Foundation of Shandong Province (grant no. ZR2020MH357, ZR2020MH312), Tai’an Science and Technology Innovation Development Project (grant no.2020NS092).

## Conflict of Interest

The authors declare that the research was conducted in the absence of any commercial or financial relationships that could be construed as a potential conflict of interest.

## Publisher’s Note

All claims expressed in this article are solely those of the authors and do not necessarily represent those of their affiliated organizations, or those of the publisher, the editors and the reviewers. Any product that may be evaluated in this article, or claim that may be made by its manufacturer, is not guaranteed or endorsed by the publisher.
